# Patterns and predictors of primary mental health service use following bushfire and flood disasters

**DOI:** 10.3402/ejpt.v5.26527

**Published:** 2014-12-09

**Authors:** Lennart Reifels, Bridget Bassilios, Matthew Spittal, Kylie King, Justine Fletcher, Jane Pirkis

**Affiliations:** Centre for Mental Health, Melbourne School of Population and Global Health, The University of Melbourne, Melbourne, Australia

**Keywords:** primary mental healthcare, access to care, service use, bushfire, flood, disasters

## Abstract

**Background:**

Mental health care services play an important role following disasters (Reifels et al., 2013). The aim of this study is to examine patterns and predictors of primary mental health care service use, following two major Australian natural disaster events.

**Method:**

Utilizing referral and session data from a national minimum dataset, descriptive and regression analyses were conducted to identify levels and predictors of the use of the Access to Allied Psychological Services (ATAPS) program over a 2-year period following two major Australian bushfire and flood/cyclone disasters. Predictor variables examined in negative binomial regression analysis included consumer (age, gender, household structure, previous mental health care history, and diagnosis) and event characteristics (disaster type).

**Results:**

The bushfire disaster resulted in significantly greater service volume, with more than twice the number of referrals and nearly three times the number of sessions. Service delivery for both disasters peaked in the third quarter. Consumers affected by bushfires, diagnosed with depression, anxiety, or both of these disorders utilized sessions at significantly higher rates.

**Conclusions:**

The substantial demand for primary mental health services following disaster can vary with disaster type. Disaster type and need-based variables as key drivers of service use intensity indicate an equitable level of service use. Established usage patterns assist with estimating future service capacity requirements. Flexible referral pathways can enhance access to disaster mental health care. Future research should examine the impact of program- and agency-level factors on mental health service use and factors underpinning treatment non-adherence following disaster.
